# ChemEx: information extraction system for chemical data curation

**DOI:** 10.1186/1471-2105-13-S17-S9

**Published:** 2012-12-07

**Authors:** Atima Tharatipyakul, Somrak Numnark, Duangdao Wichadakul, Supawadee Ingsriswang

**Affiliations:** 1 Information Systems Laboratory, National Center for Genetic Engineering and Biotechnology (BIOTEC), 113 Thailand Science Park, Phaholyothin Road, Klong 1, Klong Luang, Pathumthani, Thailand

## Abstract

**Background:**

Manual chemical data curation from publications is error-prone, time consuming, and hard to maintain up-to-date data sets. Automatic information extraction can be used as a tool to reduce these problems. Since chemical structures usually described in images, information extraction needs to combine structure image recognition and text mining together.

**Results:**

We have developed ChemEx, a chemical information extraction system. ChemEx processes both text and images in publications. Text annotator is able to extract compound, organism, and assay entities from text content while structure image recognition enables translation of chemical raster images to machine readable format. A user can view annotated text along with summarized information of compounds, organism that produces those compounds, and assay tests.

**Conclusions:**

ChemEx facilitates and speeds up chemical data curation by extracting compounds, organisms, and assays from a large collection of publications. The software and corpus can be downloaded from http://www.biotec.or.th/isl/ChemEx.

## Background

Accurate chemical data curation is essential for cheminformatics. Nowadays, researchers or exploration software can access internal or external public databases [1,2] to retrieve necessary information. Still, the major source of knowledge is scientific literature. Unfortunately, information in the literature is unstructured or semi-structured, and written in natural language. Chemical structures were embedded in reports, journals, and patents in the form of images. These cannot be input into chemical databases or chemistry software directly. Manual reproducing the information is time-consuming and liable to errors. Furthermore, rapid growth of publications results in difficulty to maintain up-to-date data sets. To overcome these problems, automatic information extraction becomes a subject of interest.

Whereas there are numerous text-mining tools in biological domain [3-6], chemical information extraction had not received attention until recently. Existing techniques for the chemical information extraction can be broadly classified into two categories: visual and textual data extraction. The visual data extraction system, such as Kekulé [7], CLiDE [8,9], chemOCR [10], OSRA [11], and ChemReader [12], focuses on interpretation of images embedded in documents while the textual data extraction focuses on mining interested entities and their relations from text. Textual data extraction is varied based on a subject domain, such as chemical names [13,14], chemical formulae [14], or drug names [15]. Information extraction from either image or text content results in missing information or semantic links between text and images. Therefore, a technique for combining two media [16] could be applied to improve knowledge discovery.

ChemEx is a software developed to assists a chemical data curation process. While it can be used with general chemical information extraction, ChemEx is designed for extracting information of natural products which are a major source of novel bioactive compounds or structures [17]. It provides a framework to integrate optical structure recognition and chemical text-mining software. The extracted information can be then visualized and exported to a database. Enormous chemical libraries become available with minimum time and effort.

## Implementation

### System overview

**Figure 1:**
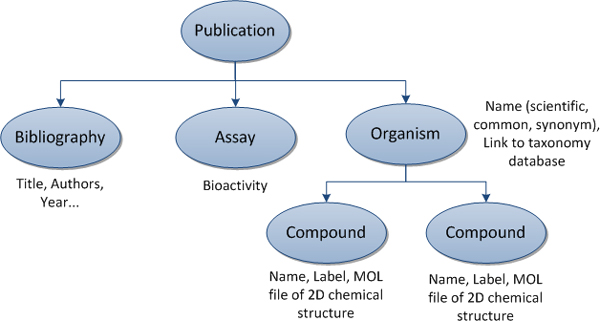
**Interested entities and their relations**.

ChemEx processes a collection of publications in order to extract information of bioactive compounds as well as an organism that produces those compounds with their bioactivity from each publication as illustrated in Figure 1. The system consists of four main modules: (a) *Document Preprocessor*, (b) *2D Chemical Structure Image Recognition*, (c) *Text Annotator*, and (d) *Information Viewer*.

**Figure 2:**
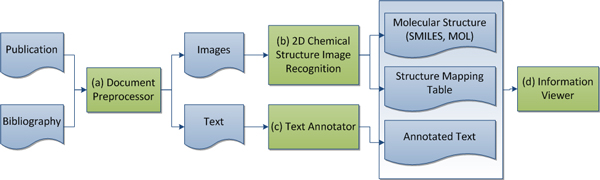
**System overview**.

Figure 2 presents the workflow of the system. First, the *Document Preprocessor *transforms and segments each input literature into textual and visual data. The 2D *Chemical Structure Image Recognition *module then translates the visual data (images) into machine readable string whereas the *Text Annotator *module tags words in a subject domain. In the end, a user can visualize extracted information using the *Information Viewer*.

### Document preprocessor module

This module pre-processes publications so that they can be input to 2D structure image recognition and text annotator modules. ChemEx works with both electronically-generated PDFs and scanned PDFs. Poppler [18] is used to segment a PDF file into a set of images and plain text. Converting full text PDF had layout errors, which are, the header and footer were mixed-up with the content, and a paragraph was sometimes broken to multiple discontinuous paragraphs. Hence, if a bibliography file is available, text content will be extracted from the abstract field in BibTeX instead. In case of the scanned PDF, which text cannot be extracted from the PDF file, BibTeX is required for the system to work properly.

It was observed that "-" is usually extracted as an unknown character "?". Therefore, for example, ChemEx replaces "Aigialomycins A?E (2?6)" with "Aigialomycins A-E (2-6)".

### 2D chemical structure image recognition module

**Figure 3:**
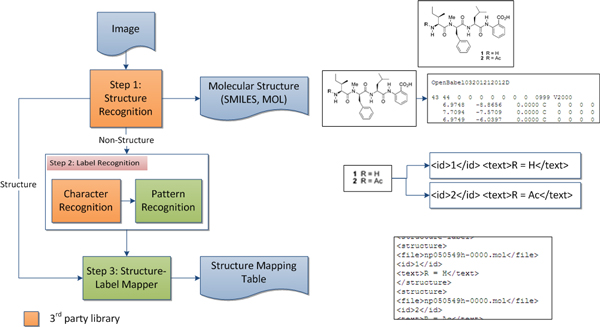
**2D chemical structure image recognition overview**.

Structure images that are embedded in publications typically consist of two parts: 2D structure of chemical molecule and label of an identifier used for referencing later in the text content. The overview of this module is illustrated in Figure 3. This module consists of three following steps: (1) *Structure Recognition *which translates each 2D image of the chemical structure into machine readable format, (2) *Label Recognition *which identifies labels in a structure image, and (3) *Structure-Label Mapper *which constructs a mapping table between the label and file location of corresponding 2D structure.

#### Step 1: structure recognition

To retrieve machine readable structure from 2D chemical structure images, ChemEx uses an open source OSRA [11]. In this step, ChemEx recovers both SMILES [19] and MDL Molfile [20] from a 2D chemical structure image. Based on OSRA features, ChemEx recognizes atomic labels and charges, circle bond (old style aromatic rings), double and triple bonds, wedge and dash bonds, and bridge bonds.

#### Step 2: label recognition

ChemEx retrieves non-structure components of the 2D structure image to identify labels of the structure. There are two parts in this step: *Character Recognition *and *Pattern Recognition*. *Character Recognition *converts non-structure image components to text using GOCR [21]. If the text pattern matches with chemical label features [16], that image component is identified as a label. ChemEx recognizes Roman digits (e.g. I, VI, X), Arabic numeral digits (e.g., 1, 2, 10), digits connected by a dash (e.g., 1-1, 3-10), digits follows by a prime (e.g., 1', VI', 1-1'), and all previous features enclosed by parenthesis (e.g., (1), (VI), (5')).

#### Step 3: structure-label mapper

One structure image may consist of multiple labels. Also, a label may contain the identification number used for reference the structure as well as others, for instance, a compound name or R-group. To construct a structure-label mapping table, ChemEx's Structure-Label Mapper assigns each 2D structure (from step 1) to a nearest label (from step 2) using minimum weight graph matching algorithm [16]. Successful mapping is written into a file to be used in *Information Viewer*.

### Text annotator module

**Figure 4:**
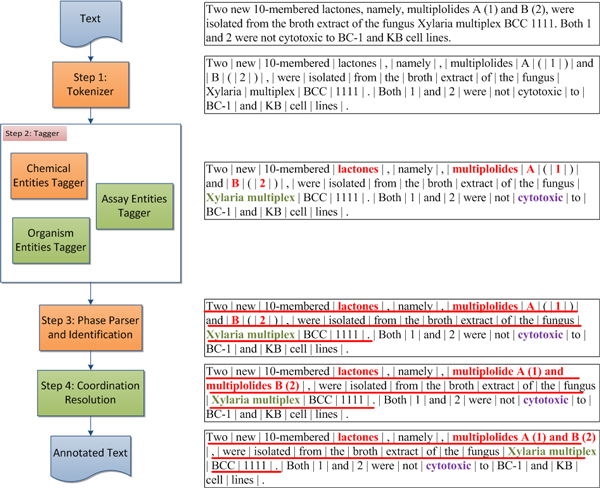
**Text annotator workflow**.

This module discovers interested entities and relations from textual information of publications. ChemEx employs a component called Analysis Engine (AE) from Unstructured Information Management Applications (UIMA) [22] to analyse document in four steps: (1) *Tokenizer*, (2) *Tagger*, (3) *Phase Parser and Identification*, and (4) *Coordination Resolution*. The processing flow among these steps is illustrated in Figure 4.

#### Step 1: tokenizer

Tokenizer splits a text stream into tokens of words. ChemEx uses the tokenizer from OSCAR4 [23] which is able to handle hyphens or other symbols in chemical terms such as 2-Amino-2-(hydroxymethyl)-1,3-propanediol hydrochloride. ChemEx also extends OSCAR's tokenizer to handle scientific name abbreviation, such as *Penicillium sp*. or *P. pacificum*.

#### Step 2: tagger

Tagger labels the interested word tokens in text. ChemEx tagger consists of *Chemical Entities Tagger*, *Organism Entities Tagger*, and *Assay Entities Tagger*.

ChemEx employs ChemicalTagger [24], which uses machine learning approach called Maximum Entropy Markov Model Recogniser [25], to (i) recognize chemical names, reaction names, enzymes, and chemistry-related terms such as experimental action verbs or units and (ii) tag general English word classes, such as a noun or a verb, which will be used in the phase parser. ChemEx uses all information from ChemicalTagger.

Organism and assay entities are tagged using dictionary-based approach. ChemEx extends ConceptMapper [26] which is a configurable dictionary UIMA-based annotator. The ConceptMapper allows a user to add or remove dictionaries according to domain of interest. The extended ConceptMapper keeps an identification number and database source so it is possible to retrieve further information of the entities. In case of organism, once scientific names, e.g., *Escherichia coli*, are detected, the tagger abbreviates all scientific names and searches the text again for abbreviated scientific names such as *E. coli*. Also, the organism tagger extends a term to cover "sp.", "spec.", or "spp." for unspecified species.

A dictionary consists of a set of entries, specified by the < token > XML tag. Each entry contains one or more variants (synonyms, common names). Taxonomic ranks (phylum, family, genus, and species) are optional. For example:

<synonym>

<token canonical="sirobasidium" id="2403010"

phylum="basidiomycota" family="sirobasidiaceae" genus="sirobasidium">

<variant base="sirobasidium"/>

</token>

<token canonical="sirobasidium brefeldianum" id="8439178"

phylum="basidiomycota" family="sirobasidiaceae" genus="sirobasidium"

species="brefeldianum">

<variant base="sirobasidium brefeldianum"/>

<variant base="sirobasidium intermediae" status="synonym"/>

</token>

</synonym>

Currently, the dictionaries of scientific names used in ChemEx are derived from Integrated Taxonomic Information System (ITIS 545,485 records, accessed on 9th December 2011) [27], List of Prokaryotic names with Standing in Nomenclature (LPSN 14,390 records, accessed on 8th December 2011) [28], and Catalogue of Life (55,022 records of fungi domain, accessed on 5th December 2011) [29]. For assay, drug-related terms from Chemical Entities of Biological Interest (ChEBI) ontology (164 records, accessed on 13th February 2012) [30] were used.

#### Step 3: phase parser and identification

ChemicalTagger [24] also parses and identifies a sentence. Phase parser receives tagged token stream and builds grammatical structure based on predefined grammars. After text is parsed, experimental action phases, such as "Compound 1 was *added *to the solution" or "Compound 1 was *extracted *from compound 2", can be identified by analysing the grammatical structure. Numbers used for compound referencing (labels) are also identified in this step. Finally, ChemEx extracts natural products and their source organism from a ChemicalTagger's "yielded" phase, such as "Compound 1 was *isolated *from the fungus *Xylaria multiplex*".

#### Step 4: coordination resolution

Sometimes, especially in an abstract, multiple compounds appear in one sentence. The compounds are joined with punctuation marks or coordinate conjunctions. Exploring semantic meaning of a noun group of compounds thus improves knowledge discovery. Coordination resolution is to identify each compound in a compound chunk mentioned in text content. For example, "multiplolides A (1) and B (2)" consists of two compounds: multiplolide A, labeled as 1, and multiplolide B, labeled as 2. "drechslerines C-G (6-10)" consists of five compounds: drechslerine C (6), drechslerine D (7), drechslerine E (8), drechslerine F (9), and drechslerine G (10).

**Figure 5:**
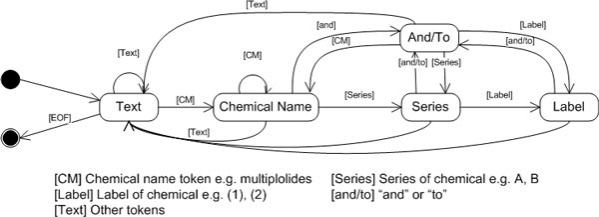
**State machine diagram for coordinate resolution**.

ChemEx uses a state machine (Figure 5) to recognize and interpret a compound group taking into account a label and series. The state machine processes on tagged token stream. *Text *state disregards non-chemical entity tokens. *Chemical Name *state accumulates a chemical name, either single or multiple words. *Series *and *Label *states are responsible for series and label token respectively. They also insert values in between two letters or numbers. For instance, "A-C" becomes "A, B, C", and "1-3" becomes "1, 2, 3". *And/To *state handles "and" and "to" token. For instance, "compounds A and B" becomes "compound A, compound B", and "compounds A to C" becomes "compound A, compound B, compound C". In the end, individual chemical names with series and label are generated as chemical entities.

### Information viewer module

**Figure 6:**
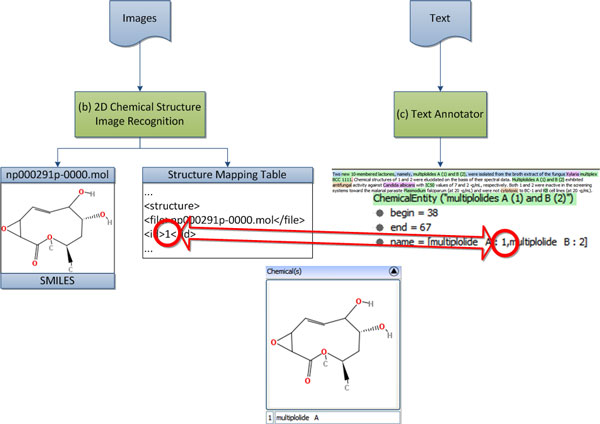
**Structure-label mapping tables usage**.

Information viewer provides graphical interface to user for viewing the integrated results from all modules. ChemEx summarizes natural products and their bioassay tests reported in a publication. The viewer includes UIMA CAS Annotation Viewer [22] to display annotated text and JChemPaint [31] to reproduce structure thumbnails from MOL files generated by *2D Chemical Structure Image Recognition *module. Additionally, structure-label mapping tables generated by *2D Chemical Structure Image Recognition *module is combined with chemical compound entities extracted from *Text Annotator *module as illustrated in Figure 6. Therefore, a chemical compound entity can be viewed and searched with its 2D chemical structure image and SMILES. A user can use the viewer to visualize results and export those results to an XML file which can be imported to sMOL Explorer [32], a web-enabled database and exploration tool for Small MOLecules datasets.

## Results and discussion

**Figure 7:**
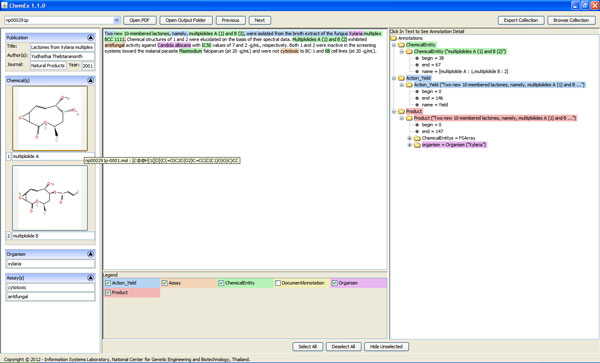
**Example of an information viewer for one document**. This main screen displays extracted information from a publication. The user can step through a collection via control buttons and export the collection to one single XML file. Structure information can be investigated further by clicking a 2D chemical structure image.

**Figure 8:**
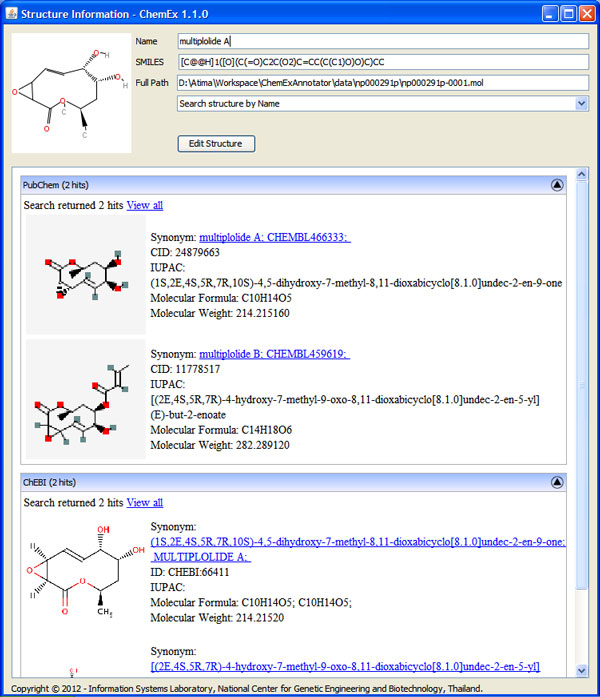
**Example of structure information from external databases**. This screen displays a structure name, SMILES, and path to the structure file extracted from a publication. The user can edit the structure file using JChemPaint. Furthermore, ChemEx uses extracted information to search through external databases via web services. The user can view the retrieved information from PubChem and ChEBI.

**Figure 9:**
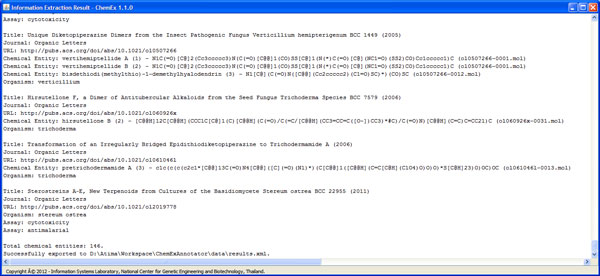
**Example of an information viewer for a collection**.

ChemEx is able to extract compound, organism, and assay entities from text content automatically. It also finds 2D chemical structure of each compound from images embedded in full text, and converts 2D chemical structure images to machine readable format. Results from ChemEx can be visualized through the information viewer as demonstrated in Figure 7. A user can view annotated text together with publication information, compound list, organism that produces those compounds, and assay tests. Each compound can be also searched for additional information from external databases [2,30] as well as edited by 2D chemical structure editor (Figure 8). Moreover, a user can view and export extracted information of all publications in a collection in one place (Figure 9).

The system was tested using literatures from ACS Publications (accessed on 13th March 2012) [33]. The keywords used for literature retrieval were "fungus Thailand". All accessible research articles with the abstract and full text PDF were downloaded. In total, 89 publications were obtained, but the test set contained only 74 publications that reports compounds with 2D chemical structures.

Each full text was retrieved with its bibliography. All images, including but not limited to 2D chemical structure images, were extracted from each PDF. Accuracy of information extraction and 2D chemical structure image recognition were evaluated.

### Information extraction evaluation

Extracted information from text content, consisting of compounds, organisms, and assays were listed by the system and compared with manually listed entities. The results are shown in Table 1. Note that entities were evaluated regardless of natural products or not.

**Table 1:** Extracted information from text content of the test set

	Exact Matches	Partial Matches	False Positive	False Negative	Precision	Recall
Compounds	203	15	41	105	83.20%	62.85%
Organisms	91	21	3	5	96.81%	77.78%
Assays	80	0	0	15	100.00%	84.21%

The test set consisted of 89 publications with terms "fungus Thailand" from ACS Publications. Only 74 publications reported compounds with 2D chemical structures. Compounds, organisms, and assays were extracted from text content and compared with manually listed entities.

An exact match was an extracted entity matching the whole term of a manually listed entity, whereas a partial match was an extracted entity matching some part of a manually listed entity. False positive (FP) was an unexpected result. False negative (FN) was a missing result. The exact match was defined as true positive (TP). By default, the partial match was classified as false negative. Precision and recall were defined as:

$$\text{Precision}=\frac{\text{TP}}{TP+FP},$$

$$\text{Recall}=\frac{\text{TP}}{TP+FN}.$$

#### Compound entities

The main purpose of the experiment was to extract compound entities from a content of abstracts and discover their 2D depiction from images embedded in full text. Thus, only compounds that have 2D structure images were considered. Partial matches were considered as mismatches.

The system extracted compound entities with 83.20% precision and 62.85% recall. ChemicalTagger achieved 61.34% precision and 22.60% recall. As demonstrated in Table 2, ChemEx increases precision and recall 21.85% and 40.25% respectively.

**Table 2:** Extracted chemical entities from text content of the test set compared to ChemicalTagger

	Exact Matches	Partial Matches	False Positive	False Negative	Precision	Recall
ChemicalTagger	73	54	46	196	61.34%	22.60%
ChemEx	203	15	41	105	83.20%	62.85%
					+21.85%	+40.25%

The main improvement resulted from ChemEx's *Coordination Resolution*, which, for example, recognized five instead of one compounds from "drechslerines C-G". The improvement could be smaller in case of full text where each compound is written separately.

Currently, *Coordination Resolution *recognizes a label that contains pure digits (i.e. 0, 1, 2). Future development could extend coordination resolution to recognize other types of label such as Roman digits or letters.

#### Organism entities

Organism recognition showed good performance with 96.81% precision and 77.78% recall. False negative were scientific names outside the domain of interest. Partial matches were scientific names that only a genus was detected without species. If partial matches were considered as true positive, the performance is up to 97.39% precision and 95.73% recall.

While dictionary-based text mining yields high precision, its recall may be low depending on dictionary size. However, large dictionaries increase processing time and memory usage. It is recommended to supply dictionaries according to the domain of interest.

#### Assay entities

Assay recognition achieved 100.00% of precision and 84.21% of recall. False negative was due to assay terms does not exist in the corpus.

Currently, ChemEx recognized only one-word assay, such as, "antifungal" or "cytotoxic". However, some assays were reported in a sentence, for example, "Compound 1 inhibited activity against the malarial parasite *Plasmodium falciparum*". Future development could apply phase parsing to recognize these assay phases.

### 2D chemical structure image recognition evaluation

Compounds entities in publications were manually listed and used to search for corresponding chemical structure in PubChem [2]. The search was done automatically via web service and the most similar of each chemical structure was used in the evaluation. In total 204 structures were found and downloaded as the ground truth. Then CACTVS script [11,34] evaluated structure similarity between ground truth and regenerated structures based on standard InChI [35].

ChemEx was able to map 144 structures (70.59%) to compound entities extracted from text content. Mapping error comes from imperfect image segmentation, OCR errors, and incomprehensive pattern in label recognition. Table 3 shows number of structures according to similarity score. "T > 70%" indicates the number of structure with similarity above 70%. There were 72 structures (35.29%) with the similarity score is above 70%. The average similarity of these 72 structures was 91.42%. ChemEx reconstructed 28 identical structures (13.73%). The average similarity between ground truth and regenerated structures was 71.86%.

**Table 3:** Results of 2D chemical structure image recognition on the test set

Thresholds Number of	Structures (% to the total)	Average Similarity Score
Cannot find the InChI	9 (4.41%)	-
T > 70%	72 (35.29%)	91.42
T > 80%	61 (29.90%)	94.43
T > 90%	44 (21.57%)	98.30
Identical structure	28 (13.73%)	100.00
Total mapped structure	144 (70.59%)	71.86

CACTVS script computed structure similarity between ground truth and regenerated structures based on standard InChI. In total 204 structures from PubChem were downloaded as the ground truth.

Our experiment found that sometimes OSRA [11] recognized a graph as chemical structure. Image classification prior to *2D Chemical Structure Image Recognition *could improve accuracy and performance. Another major issue is that OSRA interests only structure images. Retrieving non-structure image components from OSRA may result in high segmentation error, which causes error in structure-label mapping. Future development could apply segments categorization [16] before using OSRA to cover this issue.

## Conclusions

ChemEx automatically discovers chemical knowledge from a large collection of publications. It is built on top of multiple pieces of software [11,22,24] allowing information extraction from both visual and textual content. The system extracts compound, organism, and assay information with flexible framework. A user can add new dictionaries to customize results according to the domain of interest. ChemEx information viewer integrates and visualizes results. To the best of our knowledge, ChemEx is the first system that provides these functionalities. Although the accuracy needs to be improved, ChemEx increases information understanding and assists a user on chemical data curation process. We believe it is one step towards fully automatic chemical data curation, which is useful for constructing large chemical structure libraries.

## Availability and requirements

• Project name: ChemEx - Chemical Information Extraction.

• Project home page: http://www.biotec.or.th/isl/ChemEx.

• Operating system(s): Windows and Linux.

• Programming language: Java and C++.

• Other requirements: at least 2 GB of RAM. Other dependencies were listed in the home page.

• License: GNU GPL.

## Competing interests

The authors declare that they have no competing interests.

## Authors' contributions

AT developed the system and the manuscript. SN was responsible for building project homepage and software installer. SI and DW designed, supervised the project, and refined the manuscript. All authors read and approved the final manuscript.
